# The barrier and protective functions of intestinal mucin in defense against *Candida albicans*

**DOI:** 10.3389/fmicb.2025.1561004

**Published:** 2025-11-03

**Authors:** Yuanyuan Liu, Dongmei Li, Li Ma, Yiyang Wen, Dongmei Shi

**Affiliations:** ^1^The Second Clinical Medical College, Shandong University of Traditional Chinese Medicine, Jinan, Shandong, China; ^2^Department of Microbiology and Immunology, Georgetown University Medical Center, Washington, DC, United States; ^3^The Laboratory of Medical Mycology, Jining No.1 People’s Hospital, Jining, Shandong, China; ^4^Department of Pathology, Jining No.1 People's Hospital, Jining, Shandong, China; ^5^Department of Dermatology, Jining No.1 People’s Hospital, Jining, Shandong, China

**Keywords:** *Candida albicans*, mucus, mucin, glycan, barrier

## Abstract

*Candida albicans* (*C. albicans*) is an opportunistic fungal pathogen that typically colonizes intestinal mucosal surfaces asymptomatically. However, dysbiosis or disruption of the mucosal barrier can trigger its overgrowth, leading to mucosal infections and, in severe cases, systemic disease. Intestinal mucins, the main components of mucus, play critical roles in host defense by suppressing filamentation-associated gene expression, blocking the yeast-to-hypha transition, and inhibiting key virulence traits including adhesion, biofilm formation, and secretion of hydrolytic enzymes. While their function as physical barriers is well established, the molecular mechanisms underlying mucin–fungus interactions remain incompletely understood. By targeting the virulence rather than the viability of *C. albicans*, mucins offer potential advantages over conventional antifungal therapies, including limiting drug resistance, improving biofilm penetration, and preserving mucosal homeostasis. This review highlights recent advances in understanding the biological and immunological roles of mucins in modulating *C. albicans* colonization and infection, and discusses their promise as novel therapeutic and diagnostic agents in intestinal candidiasis.

## 1 Introduction

The mammalian gut harbors trillions of microorganisms, including viruses, bacteria, fungi, and parasites (Cheng et al., 2019; Jandhyala et al., 2015; Zheng et al., 2020). These microbes coexist symbiotically with host, with contributing to nutrient absorption and vitamin synthesis, while also playing a critical role in maintaining immune homeostasis (Larabi et al., 2020). At the frontline of host defense, mucosal epithelial cells as frontline, play a crucial role in defending against external threats (Linden et al., 2008) through secretion of a range of defensive factors, including mucins, antibodies, defense proteins, proteins, aggregins, cathelicidins, lysozyme, histatins, and nitric oxide (Linden et al., 2008). Collectively, these molecules establish both physical and chemical barriers with antimicrobial activity. Together with immune cells such as leukocytes, they help eliminate foreign irritants and invading microbes (Linden et al., 2008).

*Candida albicans* is a common fungal resident of human mucosal surfaces and can colonize the entire gastrointestinal tract, from the oral cavity to the colon, as well as the respiratory and reproductive mucosa under physiological conditions (Macias-Paz et al., 2023; Valentine et al., 2025). However, under pathological circumstances, such as microbiome disruption or immunosuppression, its pathogenic potential can be triggered, leading to invasive infection (Gouba and Drancourt, 2015; Kullberg and Arendrup, 2015). Multiple virulence factors contribute to its ability to cause disease, including the transition to filamentous forms, biofilm formation, adhesin expression, and the secretion of hydrolytic enzymes (Calderone and Fonzi, 2001; Takagi et al., 2022).

The mucus layer represents the first line of defense at mucosal surfaces (Pelaseyed et al., 2014). In the gut, mucus is a slippery, viscous secretion produced continuously by goblet cells (Pelaseyed et al., 2014). It serves as both a physical barrier that prevents microbial intrusion and a biochemical shield enriched with defensins, secretory IgA, and antimicrobial enzymes capable of neutralizing pathogens (Hansson, 2020). Beyond protection, mucus also functions as an ecological niche that shapes microbial survival and interactions with immune system, thereby playing a pivotal role in maintaining gut homeostasis and resisting pathogenic infections (Linden et al., 2008).

This review highlights the dual roles of mucus as both a protective barrier against *C. albicans* invasion and as a habitat and energy source for microbial community’s establishment. We examine how mucus and its key structural components, the mucins, inhibit *C. albicans* virulence, and we discuss the influence of other gut microorganisms on its colonization and invasive potential. A deeper understanding of these interactions will not only clarify the biological and immunological roles of intestinal mucus but may also open avenues for novel therapeutic strategies against invasive *C. albicans* infections.

## 2 The physicochemical barrier role of the gut mucin against *Candida albicans*

### 2.1 The mucus barrier in small intestine and colon

The organization of the intestinal mucus barrier differs between the small intestine and the colon. In the small intestine, mucus forms a single, less dense layer, whereas in the colon it is organized into two distinct layers: a dense, adherent inner layer and a loose outer layer (Hansson, 2020; Weersma et al., 2020). Despite their structural differences, the two layers share a similar composition. Indeed, the outer layer is derived from the inner layer as viscous substances gradually loosen over time (Johansson et al., 2008), highlighting the dynamic nature of the mucus barrier.

Two mucus layers in the gut perform specialized functions. The loose outer mucus layer provides a permissive niche for commensal microorganisms, promoting their colonization and contributing to the maintenance of a balanced gut microbiome. This layer is also enriched with antimicrobial peptides (AMPs) secreted by intestinal epithelial cells and Paneth cells, which form a bactericidal gradient that protects the underlying epithelial cells from pathogenic intruders (Birchenough et al., 2023; Díaz-Puertas et al., 2023; Ermund et al., 2013). The AMPs family is thought to be particularly released from cells undergoing infection-induced apoptosis, and they exert their microbicidal activity primarily by directly targeting microorganisms (Díaz-Puertas et al., 2023). In contrast, the dense inner mucus layer, which is in immediate contact with the intestinal epithelium, acts as the first line of defense and is constantly consumed and replenished (Linden et al., 2008).

Organisms within a healthy microbiota rarely penetrate the inner mucus layer, as doing so may compromise the ecological balance needed to maintain immune homeostasis (McGuckin et al., 2011). When *C. albicans* colonizes the mouse small intestines, which has a single mucus layer, the majority of fungal cells typically remain within the lumen and are found adjacent to the mucus layer. Occasionally, a small number of *C. albicans* cells can be observed in direct contact with host epithelial cells (Eckstein et al., 2020). In the colon, which has two mucus layers, approximately half of the *C. albicans* cells are located in the outer mucus layer, near the lumen side. Interestingly, the specific microenvironment occupied by fungi in the colon largely depends on the presence of individual bacterial species in the outer mucus layer. For example, *Bacteroid thetaiotaomicron* promotes mucin production, thereby supporting the growth of *C. albicans* (Eckstein et al., 2020).

Beyond its physicochemical barrier role, which physically separates *C. albicans* from the gut epithelium, mucus also actively participates in other functions, such as providing lubrication and hydration to the intestinal lining. It also serves as a key innate defense mechanism that protects the host from infection (Alabssawy et al., 2024; Valle Arevalo and Nobile, 2020). Mucus mixes with secretions from Paneth cells and intestinal epithelial cells, such as RegIIIγ, which are rich in antimicrobial peptides and lysozymes. This combination establishes an antimicrobial gradient within the mucus, helping to keep both bacteria and *C. albicans* away from the surface of epithelial cells (Pelaseyed et al., 2014; Johansson et al., 2008).

### 2.2 Mucin subfamilies

The mammalian intestinal mucus layer is an elastic gel layer primarily composed of water, electrolytes, lipids, and large glycoproteins called mucins (Ambort et al., 2012; Peterson and Artis, 2014; Sauvaitre et al., 2021). As it moves down the intestines from the small to large intestines, the composition, structure, and spatial distribution of mucoproteins are significantly changed. The thin mucus layer of the small intestine permits transient adhesion of small numbers of commensal bacteria, ensuring nutrient absorption while limiting pathogen retention; the thick mucus layer of the large intestine selectively recruits beneficial bacteria and repels pathogens through distinct terminal modifications of the O-glycan chain (high fucosylation or high sialylation), forming a positive feedback mechanism that reinforces the barrier function (Johansson et al., 2008).

These mucins, primarily secreted by goblet cells and to a lesser extent by Paneth cells, are classified into three subfamilies: secreted gel-forming mucins, secretednon-gel-forming mucins, and cell surface membrane-bound mucins (Paone and Cani, 2020; Sauvaitre et al., 2021) (Supplementary Table S1). Mucins are heavily glycosylated, with O-linked oligosaccharide side chains attached to their protein core, which contributes to the highly viscoelastic nature of mucus and barrier properties (Bakshani et al., 2018). This high degree of glycosylation enhances the adhesive and gel-like nature of the mucus (Basmaciyan et al., 2019), effectively preventing pathogens penetration while allowing the exchange of oxygen, carbon dioxide, nutrients and metabolites. Thus, mucins function both as a protective barrier and as a lubricant for the intestinal surface (Bakshani et al., 2018).

#### 2.2.1 Secreted gel-forming mucins

The thickness of the mucus layer is continuously maintained through a process of removal and regeneration, known as abscission, which efficiently separates contaminants from underlying tissues and provides defense (Bakshani et al., 2018; Cone, 2009). Secreted gel-forming mucins, including MUC2, MUC5AC, MUC5B, MUC6, and MUC19 (Supplementary Table S1), constitute the major portion of the mucin family (Paone and Cani, 2020). Their secretion is regulated by two pathways. Under physiological conditions, the constitutive pathway ensures continuous mucus secretion for mucosal maintenance, whereas a second regulatory pathway responds to environmental or pathological stimuli by rapidly releasing large quantities of mucin (de Repentigny et al., 2000). Regular renewal of the mucosal barrier benefits the host by expelling microorganisms including *C. albicans*. MUC2, largely synthesized by intestinal goblet cells in the epithelial layer, is the largest gel-forming mucin in the intestine and plays a vital role in lubricating the epithelium and facilitating molecule transport (Basmaciyan et al., 2019). In the distal colon, thedense MUC2 polymers in inner mucus layer physically limits microbial access to the epithelium, providing a protective barrier against microorganisms such as *C. albicans* (Takagi et al., 2022).

#### 2.2.2 Cell surface membrane-bound mucins

Cell surface membrane-bound mucins, MUC1, MUC3A/B, MUC4, MUC12, MUC13, MUC15, MUC16, MUC17, MUC20, MUC21, and MUC22 (Supplementary Table S1), are key components of the glycocalyx across mucosal tissues. The intestinal epithelial glycocalyx is a dense, complex, villous carbohydrate structure covering the apical membrane surface of intestinal epithelial cells (Johansson et al., 2008). It is composed of countless highly glycosylated molecules (Linden et al., 2008). Its integrity is crucial for maintaining intestinal homeostasis, preventing infection, avoiding inflammation, and ensuring nutrient absorption (Johansson et al., 2008). Disruption of the glycocalyx barrier function leads to increased intestinal epithelial permeability, making it easier for pathogens and toxins to come into contact with and damage epithelial cells, triggering inflammatory responses, and potentially causing intestinal microbiota imbalance (Linden et al., 2008; Pelaseyed and Hansson, 2020).

Among these glycocalyx proteins, MUC1 acts as a decoy to limit microbial adhesion, a function demonstrated in the mouse gastric epithelium (Pelaseyed and Hansson, 2020). MUC1 is present on the parietal surface and surface epithelium of gastric deep fossa cells (McAuley et al., 2007), where it reduces acute and chronic *H. pylori* colonization by preventing the bacterium from binding directly to enterocytes (Pelaseyed and Hansson, 2020). Additionally, mice infected with the bacterial pathogen *Campylobacter jejuni* via the oral route can upregulate the level of MUC 1 protein in the gastrointestinal tract, thereby inhibiting the pro-apoptotic effect of *Campylobacter jejuni* toxins (McAuley et al., 2007). Transmembrane mucins, such as MUC3, MUC12, and MUC17, extend approximately1μm from the brush border and form part of enterocyte glycocalyx. Interestingly, MUC17 mucins can translocate from the cell surface into intracellular vesicles, suggesting that enterocytes actively manage and respond to microbial challenges at the epithelial interface (Pelaseyed et al., 2014). Acting as a bait ligand for bacterial and fungal adhesins, MUC17 not only blocks pathogen binding but also rapidly upregulates its cytoplasmic domains to enhance cytoplasmic localization. This process limits the attachment of pathogens to other cell surface molecules and subsequent invasion (McAuley et al., 2007).

#### 2.2.3 Secreted non-gel-forming mucins

Secreted non-gel-forming mucins, such as MUC7 and MUC8 (Supplementary Table S1), contribute to microbial binding and exhibit anti-inflammatory properties but do not affect the viscoelasticity of the mucosal layer (Puri and Edgerton, 2014). MUC7, in particular, has demonstrated fungicidal activity through the histone-like structural domain found in its N-terminal region (Cha and Song, 2018). Its amphiphilic helix structure creates hydrophilic and hydrophobic surfaces, a feature required for antimicrobial activity. Moreover, a low molecular weight MUC7-20-mer fragment found in human salivary mucin can penetrate fungal cell membrane and accumulate intracellularly. This action results in nearly 90% killing efficacy against *C. albicans* at 4 °C (Bobek and Situ, 2003).

The overall functional diversity and dynamic properties of these mucins, as outline in Supplementary Table S1, are essential for maintaining the integrity and protective capabilities of the intestinal mucus layer.

### 2.3 Utilization of mucins by symbiotic microorganisms

Mucins contain a long structural domain, known as the PTS (proline-threonine–serine) domain. This domain is often heavily glycosylated through GalNAc (N-acetyl galactosamine) bonds attached to serine and threonine residues (Lang et al., 2007; Ravcheev and Thiele, 2017). The extensive glycosylation of mucins has multiple O-glycan epitopes that serve as attachment sites for symbiotic bacteria, as seen with glycosylated MUC2. MUC2 is a large mucoprotein, approximately 2.5 mDa in size, with 80% of its mass consisting of carbohydrates (Lang et al., 2007). Each MUC2 monomer provides over 3,300 terminal sugar residues through more than 1,600 O-glycans and 30 N-glycans, supporting of attachment of symbiotic bacteria (Luis and Hansson, 2023).

In situation where carbon sources are limited, glycans in mucus can be utilized by gut microbiota as an essential source of carbon for energy production (Ravcheev and Thiele, 2017). The high rate of synthesis and secretion of human intestinal mucus, along with constant flow of nutrients into the gut, creates a new ecological niche and a direct source of nutrients for gut symbiotic microorganisms (Luis and Hansson, 2023) that are capable of degrading glycans (Bakshani et al., 2018). For example, *Clostridium difficile (C. difficile)* exhibits accelerated growth in culture media supplemented with mucus derived from intestinal epithelial cells and when mucus is the only source of carbohydrates (Furtado et al., 2024). The degradation of glycans that bacteria bonds for carries out by glycoside hydrolases (GHs) in these bacteria. For example, the EatA protease from enteric *Escherichia coli* and its homolog from *Shigella* rapidly degrade human MUC2 mucin (Luis and Hansson, 2023).

Fungal species in the gut also exploit these carbohydrate resources. *Saccharomyces cerevisiae,* a resident species of the human gut, thrives in the mucosal environment and produces 61 proteins associated with mucin degradation. By breaking down host mucus, it likely uses mucus as a primary source of carbon and nitrogen (Derrien et al., 2008; van Passel et al., 2011). Similarly, *C. albicans* is exposed to the glycans present on the MUC2 surface. Its polysaccharide-degrading enzymes, such as *Sap2-Sap6* hydrolytic enzymes, enable it to utilize the O-linked polysaccharides on mucins as a carbon source (Backhed et al., 2005; Luis and Hansson, 2023). In this context, symbiotic bacteria and fungi, including *C. albicans*, can be consideredas O-glycan degraders or mucin utilizers (Luis and Hansson, 2023).

In contrast to pathogens that disrupt the mucin polymerization network and subsequently lead to structural disruption of mucins, symbiotic microorganisms have evolved to utilize mucins by degrading O-glycans without disrupting the inner mucus barrier (Luis and Hansson, 2023). These dichotomous interactions between different gut microorganisms and mucins highlight the diverse adaptive strategies required to maintain residence in the gut mucosa.

## 3 Mucin inhibit *Candida albicans* virulence factors

*C. albicans* can grow both as unicellular budding yeast and as the hyphae. The switch from yeast to hyphae is one of the most important virulence determinants (Lash et al., 2024; Sudbery, 2011; Wang et al., 2025). Mucins have been shown to down-regulate *C. albicans* filament formation, biofilm formation, and interspecies-interaction-related pathways (Takagi et al., 2022). Hyphae formation and the expression of hyphal-associated genes mediate different virulence functions, including adhesion (*HWP1*, *ALS3*), invasion (*ALS3*), oxidative stress response (*SOD5*), protein hydrolysis (*SAP4-6*), and ferritin binding (*ALS3*) (Jacobsen et al., 2012). The upregulation of these genes increases their likelihood of invading host tissues and causing greater damage in mucosal layer (Chen et al., 2020; Ding et al., 2021).

### 3.1 Mucin down-regulates the expression of adhesion-related genes and inhibits adhesion

Hyphal-associated adhesins of *C. albicans* are essential for initiating tissue invasion through binding to host cells (Valle Arevalo and Nobile, 2020). Mutants lacking key regulators of hyphal development (e.g., *ras1Δ* or *efg1Δ*) exhibit reduced adhesion potential (Jacobsen et al., 2012). Adhesins, such as *ALS* proteins, are key virulence factors that act in conjunction with hyphae formation (Chen et al., 2020). Disruption of the *ALS1* gene decreases adhesion of *C. albicans* to the endothelial cell, whereas its overexpression increases adhesion. The N-terminal region of Als1p specifically binds to fucose-containing glycans, which are abundantly present in mucins (Donohue et al., 2011). Similar to Als1p, Hwp1p also appears to bind directly to mucins (Nobile et al., 2008; Valle Arevalo and Nobile, 2020). Interestingly, Hwp1p shares structural domains with mammalian glutaminyl transferase substrates, enabling it to form stable adhesive bonds with glutaminyl transferase (Staab et al., 1999), thereby enhancing the invasion process of *C. albicans* (Kong et al., 2016).

Mucins significantly reduce *C. albicans* adhesion to abiotic surfaces, such as polystyrene (commonly used in biofilm formation assays), as well as to human epithelial mucus surfaces, thereby promoting a planktonic state in the fungus without affecting its viability (Kavanaugh et al., 2014). When co-cultured with human colon intestinal epithelial cells *in vitro*, such as HT29-MTX that can produce natural-like mucus during proliferation, *C. albicans* demonstrated a distinct adhesion behaviors depending on the presence of mucins. Specifically, after treating HT29-MTX cells with N-acetylcysteine (NAC) to enzymatically remove the pre-formed mucins, microscopic imaging revealed a significant increase in *C. albicans* adhesion to epithelial cells (Kavanaugh et al., 2014). In contrast, cells shielded by an intact mucus layer exhibited substantially fewer adherent fungal cells (Kavanaugh et al., 2014). These findings highlight the critical role of the mucus layer in preventing *C. albicans* attachment to intestinal epithelial surfaces.

**Figure 1:**
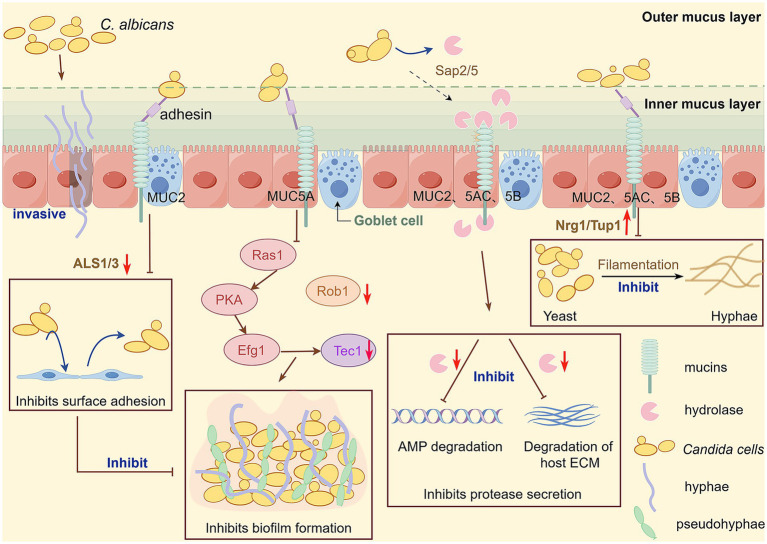
The intestinal tract of most healthy individuals is colonized with *C. albicans*, and when the balance is disrupted, *C. albicans* can break through the intestinal mucosal barrier and invade tissues and organs, causing invasive *C. albicans* infection. Intestinal mucosal barrier function plays an important role in preventing invasive infection with *C. albicans* and is essential to protect the host from pathogen infection. Mucin plays an important role in inhibiting *C. albicans* virulence factors. In the presence of mucins, several genes involved in the encoding of important virulence of *C. albicans* are downregulated, including *ALS 1* and *ALS 3* (adhesion); *Sap 5* and *Sap 2* (secretion of hydrolases); *Rob1* and *Tec1*(biofilm formation); And the transformation from yeast to hy-phal state was inhibited by upregulating *Nrg1/Tup1*. This figure is drawn by Figdraw.

Beyond the physical barrier function, mucins also exert regulatory effects at the molecular level. Studies have shown that mucins downregulate the expression of *ALS1* and *ALS3*, two key genes involved in *C. albicans* adhesion and invasion (Kavanaugh et al., 2014; Takagi et al., 2022). *ALS3* especially facilitates fungal epithelial invasion and endocytosis by binding to host cell surface E-cadherin and N-cadherin (Chen et al., 2020; Phan et al., 2023). Thus, in addition to physically entrapping *C. albicans* cells and blocking their access to the underlying epithelium, mucins appear to actively attenuate fungal adhesion by reducing *ALS3*-mediated interactions with host cadherins (Figure 1).

### 3.2 Mechanistic regulation of the yeast-to-hyphal morphological switch of *Candida albicans* by mucinoglycans

Mucins are heavily glycosylated with branched, complex oligosaccharide chains that link the anomeric carbon of the first monosaccharide to the hydroxyl group of serine or threonine residues in the mucin protein. The initial monosaccharide in this glycan chain is always GalNAc, α-linked to the mucin backbone, and subsequently extended with other sugars, including galactose, fucose, and sialic acid (Chatterjee et al., 2020). These diverse mucin glycans serve as nutrients (Tailford et al., 2015), microbial binding sites (Chatterjee et al., 2020), and signaling molecules (Bergstrom and Xia, 2013). Importantly, studies have shown that the structural complexity of mucin glycans and the mucin-rich environment contribute to maintaining *C. albicans* in its yeast form within the host (Valle Arevalo and Nobile, 2020; Kavanaugh et al., 2014).

Six glycan epitopes have been identified in MUC2, MUC5AC, and MUC5B, all derived from different combinations of GalNAc and galactose backbones that are further modified with fucose or sialic acid. Notably, four glycan structures (containing two fucose structures, one sialic acid structure, and one galactose structure) in these mucin species upregulate the transcription of the yeast-specific gene *YWP1* while simultaneously downregulating the hypha-specific gene *ECE1* (Takagi et al., 2022). These findings suggest that such glycans may play a role in suppressing the virulence of *C. albicans*.

Indeed, exposure of *C. albicans* to mucins (0.5% MUC2, MUC5AC, or MUC5B) in a methylcellulose-containing medium induced more clustered hyphae compared to the extensive mycelial cells formed in RPMI medium without these mucins (Kavanaugh et al., 2014). Real-time PCR validation demonstrated that natural mucins exhibited the strongest ability to inhibit hyphae formation (Kavanaugh et al., 2014; Soll, 2024). In the presence of mucin, *C. albicans* hyphal formation and the expression of hyphal-related genes, as well as other virulence genes, are downregulated.

In *C. albicans*, numerous proteins are involved in hyphal elongation, including *Ume6*, *Eed1*, and *Hgc1*. These proteins are co-regulated by multiple transcription factors, such as *Efg1*, *Cph1*, *Cph2*, *Czf1*, and *Flo8*, through signaling cascades, including the cAMP-dependent pathway and the MAPK pathway (Chen et al., 2020). However, mucinoglycan-mediated inhibition of filamentation appears to occur independently of both the cAMP-PKA and MAPK pathways (Takagi et al., 2022). Interestingly, the transcriptional repressor of filamentation, *Nrg1*, plays an indispensable role in this process (Wakade et al., 2024). In the presence of mucinoglycans, the transcription of several hyphal-specific genes, such as *ALS3*, *HWP1*, *EFG1*, and *HGC1*, is downregulated in wild-type strains, whereas the yeast-specific gene *YWP1* is upregulated. By contrast, in the *Δ/ΔNrg1* mutant strain, only a few genes involved in ion homeostasis were differentially down regulatedand the hyphal-associated genes suppressed in wild-type strains remain unchanged (Takagi et al., 2022). Collectively, these findings demonstrate that the downregulation of filamentation- and virulence-associated genes in response to mucinoglycans is a downstream effect of increased *Nrg1* activity during mucinoglycan-mediated inhibition of *C. albicans* filamentation (Takagi et al., 2022).

In summary, mucinoglycans interact with fungal transcription factors, particularly the repressor *Nrg1*, to prevent filament formation (Figure 1). Furthermore, mucins, through extensive glycan modifications, effectively inhibit filament formation and help maintain the yeast phase under normal physiological conditions (Takagi et al., 2022).

### 3.3 Mucins reduce *Candida albicans* biofilm formation

Microbial biofilms are communities of cells attached to solid surfaces or form at liquid-air interfaces, representing the predominant growth state for many microbial species (Davey and O'Toole, 2000; Lohse et al., 2018; Nobile and Johnson, 2015). The ability to form a robust biofilm is considered a virulence factor of *C. albicans*, as biofilm-associated cells exhibit high resistance to conventional antifungal drugs (Takagi et al., 2022). *C. albicans* biofilms can develop not only on implanted medical devices such as catheters, pacemakers, heart valves, joint prostheses, and dentures (Kojic and Darouiche, 2004; Ponde et al., 2021), but also on host surfaces, including mucosal surfaces, epithelial cell linings, and parenchymal organs (Lohse et al., 2018).

Six master transcriptional factors primarily regulate biofilm development in *C. albicans*: *Efg1*, *Tec1*, *Bcr1*, *Ndt80*, *Brg1*, and *Rob1* (Nobile et al., 2012). Additional biofilm regulators also contribute to processes such as mycelial growth (e.g., H*wp1*), extracellular matrix production (e.g., G*sc1* and *Mnn1*), and drug resistance (e.g., *Cdr1* and *Mdr1*) (Lohse et al., 2018). Studies have demonstrated that glycosylated MUC5A significantly down-regulates several key genes involving in adhesion and biofilm initiation, including *Efg1*, *Tec1*, *Brg1*, and *Rob1* (Takagi et al., 2022). In the presence of mucins, both surface coverage and biofilm thickness are reduced, with fewer cells forming hyphae within the thinner biofilm (Kavanaugh et al., 2014). In contrast to biofilms composed of yeast, hyphae, and pseudo hyphae cells on the underside of polystyrene plates in mucin-free media, the surface of the plates in the presence of mucins exhibits a single layer of yeast-like cells, with a higher proportion of cells in a nonadherent, floating state (Takagi et al., 2022). Findings suggest that mucins inhibit biofilm formation and maturation by decreasing cellular attachment to surfaces (Kavanaugh et al., 2014).

Mucus also appears to disrupt bacterial biofilms by influencing quorum-sensing pathways, as observed in *Pseudomonas aeruginosa* (*P. aeruginosa*) biofilms. Although mucins alone do not compromise the structural integrity of *P. aeruginosa* biofilms, they induce bacterial cell detachment, a process biofilm shifting cells into a less aggregated, planktonic state (Wheeler et al., 2019). This transition, potentially mediated by quorum-sensing disruption, underscores the role of mucus in modulating microbial communication and biofilm dynamics. Although it is unknown whether mucins influence quorum-sensing pathways in *Candida*, these findings provide a foundation for exploring mucus-mediated disruption of fungal biofilms and for identifying novel biofilm-targeted therapies.

In summary, mucins inhibit biofilm growth through two mechanisms: (1) inducing transcriptional responses that downregulate key biofilm-associated regulatory genes, and (2) interfering with fungal attachment and detachment processes (Figure 1).

### 3.4 Mucin down-regulates genes involved in *Candida albicans* hydrolase secretion

Three major types of extracellular hydrolytic enzymes are associated with *Candida* infections: secreted aspartyl proteinases (*Saps*), phospholipase B (PLB), and lipases (Naglik et al., 2003). Among them, the Sap family, consisting of 10 proteins, is considered a major virulence determinant, directly facilitating host tissue degradation and fungal adhesion to epithelial surfaces—both critical steps in *C. albicans* invasion. Although their functions overlap, studies have revealed subtle distinctions. For instance, *Sap1-Sap3* are primarily involved in tissue damage during superficial infections, whereas *Sap4-Sap6* play a greater role in penetrating deeper tissues and interacting with host cellular defenses. *Sap7* has been implicated in the progression of systemic fungal infections (Chen et al., 2020). Notably, elevated serum levels of *Sap9* are associated with invasive hyphal growth at infection sites, and patients infected with fluconazole-resistant *C. albicans* have been shown to produce higher levels of *Sap* proteins (Costa et al., 2010). In addition, *Sap9* and *Sap10* are essential for maintaining fungal cell wall integrity and promoting biofilm formation (Chen et al., 2020), further contributing to the pathogen’s virulence and persistence.

The proteolytic activity of Saps has also been examined in the context of mucin degradation. Sap2, a key enzyme involved in *C. albicans* adhesion to host cells (de Repentigny et al., 2000), exhibits broad substrate specificity that includes mucins. It can directly cleave the mucin backbone and glycosylated bonds (Colina et al., 1996), as demonstrated by the appearance of a “zone of clearing” within the mucin layer under electron microscopy. However, mucins can counteract this activity by significantly downregulating *C. albicans* hydrolase gene expression, including *Sap2* and *Sap5* (Kavanaugh et al., 2014), thereby enhancing the host’s defense against fungal invasion (Figure 1). Beyond its degradative capacity, *Sap2* is highly immunogenic and modulates host immune responses by degrading complement proteins (C3b, C4, and C5) and extracellular matrix components to overcome physical barriers to invasion. In murine models, infection with *Sap2*-expressing strains induces a tissue remodeling and repair–oriented immune environment characterized by macrophage polarization toward the M2 phenotype, secretion of TGF-β, and T-cell responses that collectively promote an immunosuppressive microenvironment (Lin et al., 2023). Thus, mucin-mediated repression of secreted protease genes favors host defense mechanisms. By suppressing invasive genes such as *Sap2*, mucins may inhibit early steps of fungal colonization, limiting *C. albicans* persistence at the infection site and thereby reducing the likelihood of a pronounced immune response (Kavanaugh et al., 2014).

## 4 Mucins as potential targets for clinical therapy

Current antifungal therapies are increasingly compromised by the emergence of drug-resistant *C. albicans* and their limited efficacy against biofilm-associated infections. Both challenges reduce treatment success and contribute to persistent or recurrent disease. In this context, mucins and their O-linked glycans present an intriguing opportunity as potential therapeutic or diagnostic tools for candidiasis. Could these host-derived molecules, which naturally restrict *C. albicans* adhesion, invasion, and filamentation, be harnessed to develop novel antifungal strategies?

Mucin O-glycans are synthesized by intestinal goblet cells within the Golgi apparatus, where oligosaccharide chains are covalently linked to the mucin core protein through O-glycosylation (Luis and Hansson, 2023). Subsequent extensions by fucosylation, sialylation, and sulfation collectively transform mucins into “smart gatekeepers” (González-Morelo et al., 2020). These structural modifications provide exclusive anchoring sites for beneficial bacteria, establish electrostatic barriers against pathogens, and form traps for antimicrobial peptides. Composition of mucin oligosaccharide chains varies along the gut: in the ileum, they are predominantly neutral and highly fucosylated, whereas in other regions they are more sialylated and/or sulfated. By the distal colon, the sugar chains gradually become more acidic, while the proportion of fucose residues decreases (Robbe et al., 2004).

Mucin O-glycans have shown considerable potential in the diagnosis of intestinal infections and inflammatory diseases. Using combined proteomic and glycomic approaches on high-molecular-weight proteins, certain mucin O-glycans have been identified as promising biomarkers for monitoring disease progression (González-Morelo et al., 2020). For instance, the major gel-forming mucins MUC5B and MUC5AC in the sputum of cystic fibrosis (CF) patients display a distinctive glycosylation pattern, characterized by reduced sulfation, increased sialylation, and decreased fucosylation (Schulz et al., 2007). Importantly, these glycosylation modifications and their degradation levels are consistent across CF patients, greatly strengthening their potential as reliable biomarkers for predicting pulmonary disease status (Schulz et al., 2007). This discovery offers valuable insights into evaluating *Candida* colonization or infection in the gastrointestinal tract. Systematic analysis of intestinal mucin glycosylation patterns may therefore provide a precise means of confirming infection and accurately assessing its severity.

Unlike traditional antifungal drugs that primarily eliminate pathogens, mucin O-glycans exert multifaceted antifungal effects. They maintain a commensal rather than pathogenic lifestyle within the intestinal mucosa by attenuating *C. albicans* pathogenicity through the inhibition of hyphal formation, adhesion, and biofilm production (Takagi et al., 2022). This strategy helps preserve the balance of the intestinal microbiota and maintain mucosal integrity, thereby avoiding the tissue damage often caused by conventional therapies (Luis and Hansson, 2023). In addition to their direct antifungal properties, mucins and synthetic glycans may also function as promising drug carriers for targeted antifungal therapy. Their molecular structures enable specific interactions with biofilms and the mucosal environment, thereby enhancing drug delivery to infection sites, particularly in biofilm-associated infections linked to medical devices (Wang et al., 2025). As host-derived molecules, mucin analogs are inherently biocompatible, making them suitable for long-term prophylaxis in immunocompromised patients (Wang et al., 2025). Collectively, these findings highlight the potential of mucins as innovative solutions for the comprehensive management of *C. albicans* infections.

## 5 Conclusion and perspective

*Candida albicans*, the predominant opportunistic fungal pathogen in the gut, maintains a delicate balance with host factors and the microbiome. Dysbiosis or immunosuppression can disrupt this equilibrium, triggering pathogenic activation. Mucus layer represents a critical component of the host’s innate defense system, covering all non-keratinized epithelia and providing a protective barrier against microbial intruders. Central to this defense are mucins—highly glycosylated proteins that interact with diverse microorganisms and contribute substantially to antimicrobial protection.

Unlike conventional antifungal agents that act by killing fungal cells, mucins target key *C. albicans* virulence traits, making them promising candidates for next-generation antifungal strategies. Such approaches may offer unique advantages by limiting the emergence of drug resistance, disrupting biofilm formation, and enhancing the efficacy and safety of existing antifungal therapies.

Despite systematic reviews of the *in vitro* phenomenon of mucus inhibiting *C. albicans*, the precise molecular signaling pathways mediating these inhibitory effects remain poorly defined. Mucins employ multiple strategies against *C. albicans*, including: (i) physically entrapping fungal cells to block epithelial adhesion; (ii) modulating adhesion- and hypha-associated genes (e.g., *ALS1* and *ALS3*) to restrict morphogenesis; (iii) disrupting biofilm formation by altering attachment–detachment dynamics, and (iv) suppressing initiation genes; and downregulating secreted hydrolases that are essential for tissue invasion.

Exogenous mucin supplementation has shown potential to restore barrier function and rebalance the gut microbiota in patients under critical care, with inflammatory bowel disease, or with post-chemotherapy mucositis. In these individuals, the mucus layer is often thin, exhibits disrupted glycosylation, and shows reduced mucin secretion (Cornick et al., 2015). However, therapeutic application faces several challenges. Patients frequently exhibit abnormal gastric acidity, elevated protease activity, and altered intestinal transit, all of which can degrade orally administered recombinant mucins. Alternative delivery routes (e.g., rectal or nasojejunal) encounter additional obstacles, including rapid mucus dilution, poor targeting, and competition for binding sites with microbial metabolites. These factors complicate the maintenance of effective local concentrations. Furthermore, interactions with other intestinal microbes and their metabolites may modulate the effects of mucus on *C. albicans*. Thus, the observed antimicrobial activity likely arises from a synergy between a broad-spectrum physicochemical barrier and specific immunomodulatory mechanisms.

While mucin-based therapeutics hold considerable promise against drug-resistant fungal infections, their clinical translation remains limited by several hurdles: (i) insufficient understanding of molecular mechanisms, which impedes rational drug design and resistance prediction; (ii) high production costs due to the structural complexity of natural mucins; and (iii) concerns regarding *in vivo* delivery, pharmacokinetics, and long-term safety. Importantly, excessive mucin secretion is associated with tumor progression, whereas the implications of reduced mucin expression remain controversial (Wi et al., 2021). This uncertainty suggests that mucin-based antifungal therapies should employ the lowest effective dose for the shortest necessary duration to minimize potential risks, such as reactivation of oncogenic pathways in inflamed or injured tissues. Current conclusions are largely derived from static *in vitro* co-culture models and lack validation in dynamic *in vivo* systems that reflect the three-dimensional structure of the gut mucus layer and its interplay with the immune system and microbiota. Future research should aim to bridge this gap by establishing a translational framework from mechanism to clinic. Key priorities include: (i) characterizing the binding kinetics between key mucin domains and fungal surface proteins (e.g., *ALS3*) to inform the development of small-molecule inhibitors or functional peptides; and (ii) expending beyond single-pathogen models to an ecosystem-level studies that elucidate mucins’ regulatory roles within the broader microbial community such as human-relevant models (e.g., humanized microbiota animals, intestinal organoids) to monitor microbial evolution under sustained selective pressure. Through such integrative approaches, mucin therapy may ultimately transition from conceptual frameworks to viable clinical intervention, offering new options for refractory fungal infections.
