# Supplementary material for: The barrier and protective functions of intestinal mucin in defense against Candida albicans
Source: Front Microbiol. 2025 Nov 3;16:1561004. doi: 10.3389/fmicb.2025.1561004 (PMC12620913; doi:10.3389/fmicb.2025.1561004)
Supplement: Supplementary file 1 [file Table_1.docx]

**Supplementary Table 1. Distribution of mucins and their associations to cancer and *C. albicans***

| **Mucin** | | **Distribution of mucin at the protein level in human tissue** | **Increased expression in cancer** | **Role in *C. albicans* infection** | **Reference** |
| --- | --- | --- | --- | --- | --- |
| **Secreted gel- forming mucins** | MUC2 | Small intestine, colon, rectum, doudenum, urinary bladder, urinary bladder, adipose tissue | Glioma, colorectal cancer, stomach cancer, carcinoid, pancreatic cancer, urothelial cancer, prostate cancer, breast cancer, ovarian cancer | Barrier effect.  Inhibit *C. albicans* virulence factors. | (Linden et al., 2008; Takagi et al., 2022) |
|  | MUC5AC | Nasopharynx, bronchus, stomach, gallbladder, kidney, cervix | Stomach cancer, pancreatic cancer | Barrier effect.  Inhibit *C. albicans* virulence factors. | (Linden et al., 2008; Takagi et al., 2022) |
|  | MUC5B | Nasopharynx, bronchus, salivary gland, esophagus, stomach, colon, rectum, gallbladder, cervix, endometrium, appendix | Lung cancer, colorectal cancer, stomach cancer, liver cancer, carcinoid, pancreatic cancer, breast cancer, cervical cancer, ovarian cancer | Barrier effect.  Inhibit *C. albicans* virulence factors. | (Linden et al., 2008; Sharma et al., 1998; Takagi et al., 2022) |
|  | MUC6 | Stomach, doudenum, gallbladder, pancreas, seminal vesicle | Stomach cancer, liver cancer, pancreatic cancer, prostate cancer, breast cancer, endometrial cancer, ovarian cancer |  | (De Bolós et al., 1995; Linden et al., 2008) |
|  | MUC19 | Non-expression at the protein level |  |  |  |
| **Secreted non-gel forming mucins** | MUC7 | Salivary gland |  | Exhibit fungicidal activity. (Especially *C. albicans*) | (Calderone et al., 2001; De Bolós et al., 1995; Hebbar et al., 2005) |
|  | MUC8 | Respiratory tract, middle ear epithelium |  |  | (Cha et al., 2018; Hebbar et al., 2005; Kerschner et al., 2010) |
| **Cell surface membrane-bound mucins** | MUC1 | Adrenal gland, nasopharynx, bronchus, lung, oral mucosa, salivary gland, esophagus, stomach, small intestine, colon, rectum, doudenum, gallbladder, pancreas, kidney, urinary bladder, testis, epididymis, prostate, seminal vesicle, breast, cervix, endometrium, fallopian tube, placenta, skin, bone marrow, appendix, lymph node, tonsil | Thyroid cancer, lung cancer, colorectal cancer, head and neck cancer, stomach cancer, liver cancer, carcinoid, pancreatic cancer, renal cancer, urothelial cancer, prostate cancer, testis cancer, breast cancer, cervical cancer, endometrial cancer, ovarian cancer, skin cancer | Effect *C. albicans* burden. | (Linden et al., 2008; McAuley et al., 2007; Saeland et al., 2007) |
|  | MUC3A/B | Non-expression at the protein level | Colorectal cancer, liver cancer, carcinoid, pancreatic cancer, renal cancer, prostate cancer |  |  |
|  | MUC4 | Nasopharynx, bronchus, salivary gland, stomach, small intestine, colon, rectum, doudenum, prostate, cervix, fallopian tube, appendix | Lung cancer, colorectal cancer, stomach cancer, pancreatic cancer, urothelial cancer, prostate cancer, testis cancer, breast cancer, cervical cancer, endometrial cancer, ovarian cancer, skin cancer |  | (Linden et al., 2008; Moniaux et al., 2000) |
|  | MUC12 | Colon, rectum, appendix | Colorectal cancer |  | (Linden et al., 2008) |
|  | MUC13 | Small intestine, colon, rectum, doudenum, appendix | Colorectal cancer, stomach cancer, ovarian cancer |  | (Linden et al., 2008) |
|  | MUC15 | Cerebral cortex, thyroid gland, nasopharynx, bronchus, oral mucosa, salivary gland, esophagus, stomach, small intestine, colon, rectum, doudenum, gallbladder, pancreas, kidney, urinary bladder, testis, epididymis, seminal vesicle, vagina, breast, cervix, endometrium, fallopian tube, placenta, skin, appendix, tonsil | Thyroid cancer, lung cancer, head and neck cancer, stomach cancer, liver cancer, pancreatic cancer, urothelial cancer, breast cancer, cervical cancer, ovarian cancer, skin cancer |  | (Linden et al., 2008; Pallesen et al., 2002) |
|  | MUC16 | Nasopharynx, bronchus, salivary gland, cervix, endometrium, fallopian tube | Lung cancer, head and neck cancer, stomach cancer, liver cancer, pancreatic cancer, urothelial cancer, prostate cancer, testis cancer, breast cancer, cervical cancer, endometrial cancer, ovarian cancer, skin cancer |  | (Hori et al., 2004; Linden et al., 2008) |
|  | MUC17 | Small intestine, doudenum |  |  | (Linden et al., 2008) |
|  | MUC20 | Non-expression at the protein level |  |  |  |
|  | MUC21 | Esophagus, vagina, cervix |  |  | (Li et al., 2024) |

**Supplementary Table 1: The data in this table is based on The Human Protein Atlas.**
